# CD133^+^-Derived Exosomes Carrying EIF3B Mediate Cell Metastasis and Stemness in Colorectal Cancer

**DOI:** 10.2174/0115680096346009250628215410

**Published:** 2025-07-11

**Authors:** Xiangwei Liao, Xiaodong Han, Yu Wang, Jun Yan, Zhenqian Wu

**Affiliations:** 1 Department of Gastroenterology, Shanghai Sixth People’s Hospital Affiliated to Shanghai Jiao Tong University School of Medicine, Shanghai, China;; 2 Department of General Surgery, Shanghai Sixth People’s Hospital Affiliated to Shanghai Jiao Tong University School of Medicine, Shanghai, China

**Keywords:** Exosomes, CD133+, EIF3B, colorectal cancer, intestinal neoplasms, digestive system

## Abstract

**Background:**

Colorectal cancer (CRC) is among the most widespread malignancies worldwide and is a leading cause for cancer mortality. The interstitial interaction between cancer and stem cells is important during cancer cell metastasis.

**Objective:**

In this study, we aimed to elucidate the regulatory role and the underlying mechanisms controlling the activity of exosomes derived from cancer stem cells (CSCs).

**Methods:**

Our group isolated exosomes from CSCs and non-CSCs to examine their regulatory mechanisms using Transwell migration, Cell Counting Kit-8 (CCK-8), and 5-ethynyl-2′-deoxyuridine (EdU) assays.

**Results:**

The role of Eukaryotic Translation Initiation Factor 3 Subunit B (EIF3B) in CRC was examined using an *in vivo* tumorigenesis mouse model. It was found that treatment with exosomes isolated from CD133^+^ cells (CD133+Exos) promoted the proliferation and migration of SW480 cells. The downregulation of EIF3B reduced the proliferation and migration-promoting effects of CD133^+^ Exos on SW480 cells. Furthermore, CD133^+^ Exos treatment promoted the tumorigenesis of SW480 cells.

**Conclusion:**

Our findings demonstrate that CSC-derived exosomes transport EIF3B into CRC cells to initiate epithelial-to-mesenchymal transition (EMT) and promote metastasis.

## 1 INTRODUCTION

Colorectal cancer (CRC) is among the most widespread malignancies and is a major factor contributing to cancer mortality around the globe [1, 2]. Effective clinical interventions for early-stage CRC, including surgery, chemotherapy, and radiotherapy, are generally palliative and improve patients' quality of life in the advanced stages. However, tumor recurrence and metastasis are still associated with high mortality rates [3]. Increasing evidence has demonstrated that cancer stem cells (CSCs) are present in significant numbers in tumors. CSCs are a rare, heterogeneous tumor cell population that possesses tumor initiation and self-renewal capabilities, which lead to drug resistance and ultimately treatment failure [4, 5]. Aldehyde dehydrogenase (ALDH) and its isoenzyme ALDH1, in addition to CD133, are markers found predominantly in CSCs [6]. It has been reported that many genes involved in drug resistance and tumor aggressiveness are upreg-ulated in CD133^(+)^ D10 T helper cells, and targeting them has been proven to be an efficient strategy for the treatment of melanoma [7].

Exosomes are small extracellular vesicles ranging in size from 30-100 nm, which are derived from endosomes and released by all cell types. They regulate intercellular interactions by transferring molecular contents between different types of cells. Exosomes released by cancer cells have a key function in intracellular communication involved in cancer progression [8-11]. However, the role of exosomes isolated from CSCs in CRC progression remains unclear. Here, we elucidate the regulatory role and underlying mechanisms with respect to CSC-derived exosomes in CRC.

## 2 MATERIALS AND METHODS

### 2.1 Cell culture and Transfections

The human CRC cell line SW480 (Shanghai Chinese Academy of Sciences, Shanghai, China) was cultivated in DMEM (Gibco, USA) supplemented with 1% penicillin, streptomycin (Gibco), and 10% fetal bovine serum (FBS) at 37^o^C in an atmosphere of 5% CO_2_. SW480 cells were transfected with silencing (si) RNA si-EIF3B (GenePharma, Shanghai, China) using Lipofectamine 2000 (Invitrogen, Carlsbad, CA, USA) for 2 days. We employed quantitative real-time polymerase chain reaction (qRT-PCR) to determine the transfection efficiency.

To further validate the effects of EIF3B *in vivo* experiments, a lentiviral-based small hairpin RNA (lentiviral vector: CMV-MCS-GFP-PURO; Novagen) targeting EIF3B was constructed. Green fluorescent protein detection was performed 72 h after infecting SW480 cells, and when green fluorescence exceeded 95%, the transfection was considered successful.

### 2.2 Exosome Isolation and Applications

Exosomes were isolated following the method of Wang *et al.* [12]. In brief, we starved patient-derived cells for 0.5 days without FBS. Then, we collected the medium, which we centrifuged at 300 g for 10 min, and at 20 kg for 20 min at 4°C to remove cellular debris. We then filtered the supernatant through a 0.2-mm filter, followed by centrifugation at 100×kg for 1.5 h at 4°C. The final pellet, containing exosomes, was resuspended in PBS, and the exosome number and size were determined using NanoSight. Our team quantified exosome protein content by performing the BCA protein assay. For *in vitro* exosome applications, we incubated 5 × 10^5^ cells with 50 μg of exosomes. For *in vivo* exosome applications, we injected 5 mg of exosomes intravenously into the tail vein of BALB/c nude mice (SLAC Laboratory Animal Company, Shanghai, China).

### 2.3 Sphere‐forming Assay

We plated cells or exosome‐treated cells (500 cells/well) in 6‐well plates with ultralow adherence and cultivated them in medium supplemented with B27 (Invitrogen), heparin (Sigma‐Aldrich, St. Louis, MO, USA), 20 ng/mL, epidermal growth factor (Peprotech, Rocky Hill, NJ, USA), 20 ng/mL bFGF (Peprotech), and N2 supplement (Thermo Fisher Scientific) for 3 days to allow sphere formation.

### 2.4 qRT-PCR

Our team extracted total RNA from cells using the TRIzol Reagent Kit (Invitrogen). We synthesized cDNA, which was amplified using the TaqMan Reverse Transcription Kit (Applied Biosystems, Foster City, CA, USA). qRT-PCR was performed using a TaqMan™ MicroRNA Assay Kit (Applied Biosystems). Our team employed the 2^−ΔΔCT^ approach to determine relative mRNA expression levels. *GAPDH* served as the internal reference.

### 2.5 Cell Proliferation

Cells were plated at a density of 2 × 10^3^ cells/well in 96-well plates. At various time points, the absorbance of each sample was measured at 450 nm using Cell Counting Kit (CCK-8; Yeasen Biotech Co., Ltd, Shanghai, China). We then plotted cell viability curves.

### 2.6 Transwell Migration Assay

Cells were treated with exosomes for 48 h at a dose of 2.0 × 10^5^/mL. We added 200 μL of the cell suspension into the upper chambers of Transwell inserts (Millipore, Billerica, MA, USA), while 500 μL of medium containing 10% FBS was placed in the lower chamber. After 1 day, we fixed cells that had migrated from the upper chamber to the lower surface with methanol for 15 min and stained them with crystal violet for 20 min. A microscope was used to count the number of invading cells in five randomly selected fields of view for each sample.

### 2.7 5-Ethynyl-2′-deoxyuridine (EdU) Assay

Levels of DNA synthesis and cell proliferation were assessed using an EdU Assay Kit (RiboBio, Guangzhou, China). SW480 cells were seeded at 1 × 10^4^ cells/well into 96-well plates overnight. We incubated the cells the following day with EdU solution (25 μM) for 1 day. We then fixed the cells in 4% formalin at room temperature for 2 h, followed by permeabilization with 0.5% Triton X-100 for 10 min. Our team then incubated the samples in 200 μL Apollo reaction solution for 0.5 h to stain EdU, followed by 200 μL DAPI to stain nuclei. We assessed cell proliferation and DNA synthesis using a Nikon microscope (Nikon, Tokyo, Japan).

### 2.8 *In Vivo* Experiments

Our group established a nude CRC mouse model by injecting SW480 cells (2 × 10^6^) transfected with si-negative control (NC) or si-EIF3B into the flanks of nude mice, and subsequently measured tumor size and weight. BALB/c nude mice (male), four weeks old and weighing 15~20 g, were obtained from SLAC Laboratory Animal Company, Shanghai, China. The Animal Ethics Committee of Shanghai Sixth People’s Hospital Affiliated to Shanghai Jiao Tong University School of Medicine approved all animal experiments (DWLL2022-0437). All animals were used in accordance with The US National Research Council's “Guide for the Care and Use of Laboratory Animals,” The US Public Health Service's “Policy on Humane Care and Use of Laboratory Animals,” and “Guide for the Care and Use of Laboratory Animals.” The animal experiment included NC, CD133^+^-Exo and si-CD133^+^-Exo groups, with 6 mice in each group.

### 2.9 Western Blot (WB) Assay

Protein was extracted from cells using RIPA lysis buffer. WB analysis was performed using anti-CD81 (1:1,000), anti-CD63 (1:1,000), anti-CD133 (1:1,000), anti-E-cadherin (1:1,000), anti-N-cadherin (1:1,000), and anti-GAPDH (1:1,000) primary antibodies from Cell Signaling Technology (Beverly, MA, USA). We then visualized the protein bands using the Chemiluminescence Detection Kit (Donghuan Biotech, Dongguan, China).

### 2.10 Statistical Analyses

Data are presented as the mean ± SD. GraphPad Prism (GraphPad, La Jolla, CA, USA) was used to compare differences between subgroups. P-value ≤ 0.05 was considered statistically significant.

## 3 RESULTS

### 3.1 Characterization and Measurement of Exosomes Isolated from SW480 Cells

**Fig. (1):**
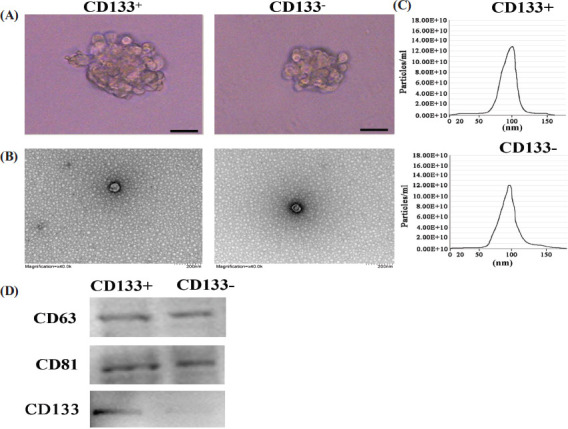
Characterization and measurement of exosomes isolated from SW480 cells. (**A**) Cell sphere formation capability in CD133^+^ cells and CD133^−^ cells was evaluated by sphere-forming assay. (**B**) Representative transmission electron micrographic images of two types of exosomes from CD133^+^ cells and CD133^−^ cells. Star bar: 20 μm. (**C**) Nanoparticle tracking shows the diameter of the particles. (**D**) WB detection shows the expression of exosome marker proteins CD63, CD81, and CD133.

First, we sought to determine whether CD133^+^ or CD133^−^ cells (isolated from SW480 cells) were associated with CRC cell stemness, metastasis, and sphere formation. We found that CD133^+^ cells had a higher cell sphere-forming capability than CD133^−^ cells (Fig. **1A**). Next, we isolated exosomes from both CD133^+^ and CD133^−^ cells and assessed exosome morphology through transmission electron microscopy (Fig. **1B**), while we determined the exosome sizes and concentrations using nanoparticle tracking analysis. We found that the diameters of our isolated exosomes were within the normal size range (Fig. **1C**). WB analysis revealed that the isolated exosomes expressed the exosome marker proteins CD63 and CD81. The results also showed that downregulation of CD133 significantly inhibited its expression (Fig. **1D**).

### 3.2 Exosomes from CD133+ Cells Promote the Proliferation and Migration of SW480 Cells

**Fig. (2):**
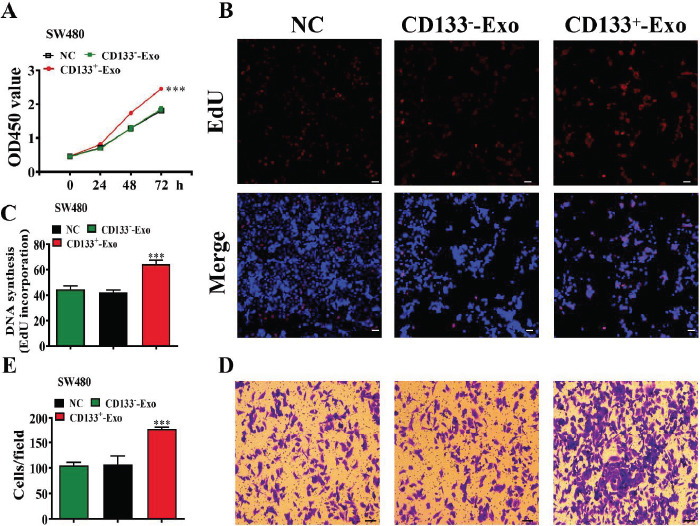
Exosomes from CD133^+^ cells promoted the proliferative and migratory capacity of SW480 cells. (**A**) CCK-8 detection shows the proliferative capacity of SW480 cells after treatment with exosomes from CD133^+^ or CD133^−^ cells. ***P<0.001 *vs* NC. (**B** and **C**) EdU detection shows the proliferative capacity of SW480 cells after treatment with exosomes from CD133^+^ or CD133^−^ cells. ***P<0.001 *vs* NC. Star bar: 20 μm. (**D** and **E**) Transwell detection shows the migratory capacity of SW480 cells after treatment with exosomes from CD133^+^ or CD133^−^ cells. ***P<0.001 *vs* NC. Star bar: 20 μm.

The effects of treating SW480 cells with exosomes derived from CD133^+^ and CD133^−^ cells were examined using cell proliferation and migration assays. The CCK-8 (Fig. **2A**) and EdU (Fig. **2B** and **2C**) assays revealed that exosomes from CD133^+^ cells (CD133^+^Exos) promoted higher levels of cellular proliferation than exosomes from CD133^-^ cells (CD133^-^Exos). Similarly, increased migration was observed following treatment with CD133^+^Exos compared to CD133^-^Exos, as measured by the Transwell assay (Fig. **2D** and **2E**).

**Fig. (3):**
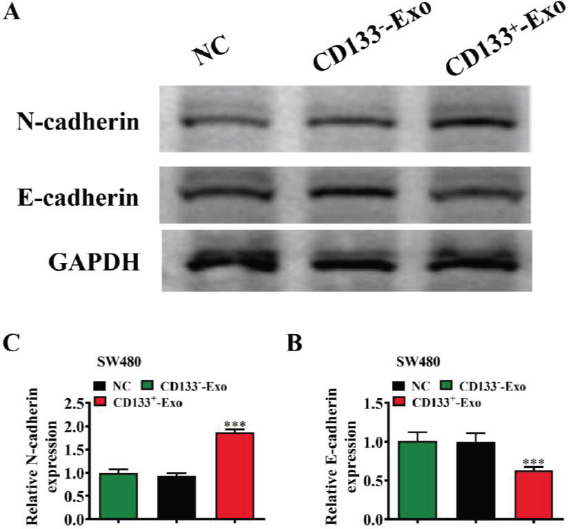
Treatment of SW480 cells with exosomes from CD133^+^ cells promoted the EMT of SW480 cells. (**A**-**C**) WB detection shows the expression of EMT-related proteins E-cadherin and N-cadherin. ***P<0.001 *vs.* NC.

WB data demonstrated that treatment of SW480 cells with exosomes from CD133^+^ cells led to increased N-cadherin expression and reduced E-cadherin expression (Fig. **3**), suggesting that CD133^+^Exo treatment promoted epithelial-to-mesenchymal transition (EMT).

### 3.3 Downregulation of EIF3B Reduces the Proliferation- and the Migration-promoting Effects of CD133+Exo Treatment on SW480 Cells

**Fig. (4):**
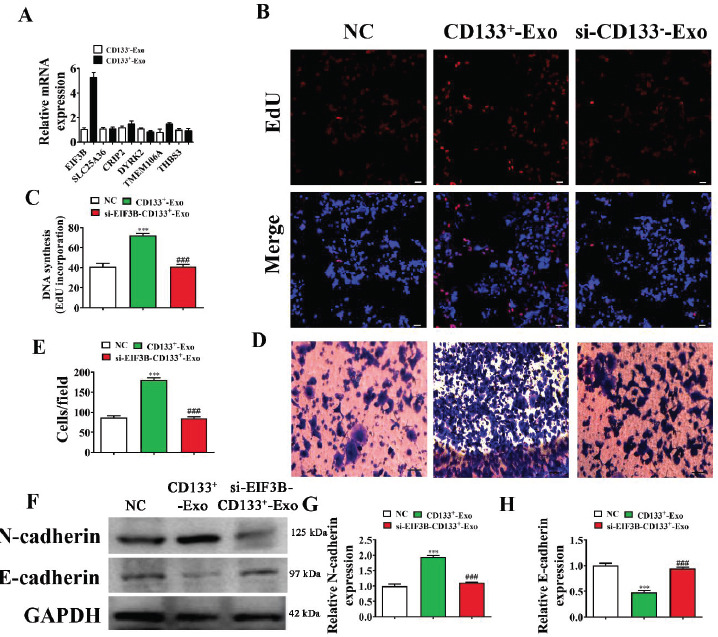
Downregulation of EIF3B decreased the promotional effect of CD133^+^ cell exosomes on SW480 cell proliferation and migration. (**A**) qRT-PCR detection shows the expression of mRNA between exosomes from CD133^+^ cells and CD133^−^ cells. (**B** and **C**) EdU detection shows the proliferative capacity of SW480 cells after treatment with exosomes from CD133^+^ or CD133^−^ cells. ***P<0.001 *vs.* NC. ^###^*P*<0.001 *vs.* CD133^+^-Exo. Star bar: 20 μm. (**D** and **E**) Transwell detection shows the migratory capacity of SW480 cells after treatment with exosomes from CD133^+^ or CD133^−^ cells. ***P<0.001 *vs.* NC. ^###^*P*<0.001 *vs.* CD133^+^-Exo. Star bar: 20 μm. (**F**-**H**) WB detection shows expression of EMT-related proteins E-cadherin and N-cadherin. ***P<0.001 *vs.* NC. ^###^*P*<0.001 *vs*. CD133^+^-Exo.

Using qRT-qPCR, we found that the mRNA expression levels of EIF3B were significantly increased in exosomes from CD133^+^ cells compared to exosomes from CD133^-^ cells (Fig. **4A**). The downregulation of EIF3B decreased the proliferation-promoting effects of CD133^+^Exos on SW480 cells, as measured by the EdU assay (Fig. **4B** and **4C**). Likewise, Transwell data revealed that EIF3B downregulation decreased the migration-promoting effects of CD133^+^Exos on SW480 cells (Fig. **4D** and **4E**). Finally, WB analysis demonstrated that downregulation of EIF3B decreased the EMT-promoting effects of CD133^+^Exos as indicated by changes in E-cadherin and N-cadherin expression levels (Fig. **4F**-**4H**).

### 3.4 CD133+Exo Treatment Promotes the Tumorigenesis of SW480 Cells

**Fig. (5):**
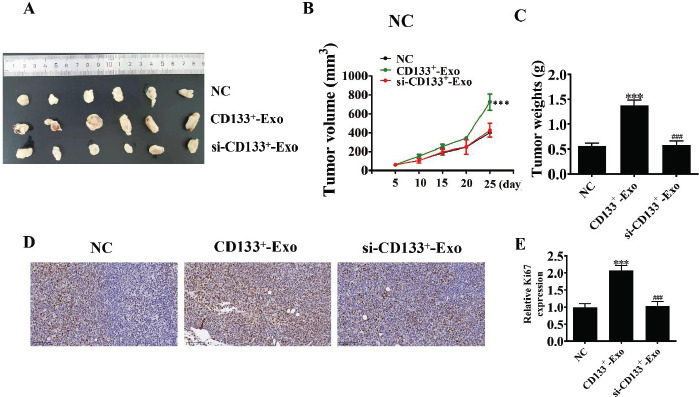
Exosomes from CD133^+^ cells promoted the tumorigenesis of SW480 cells *in vivo*. (**A**) Representative tumors excised from xenografts in nude mice implanted with SW480 cells. (**B** and **C**) Tumor volume and weight were detected. ***P<0.001 *vs* NC. ^###^*P*<0.001 *vs* CD133^+^-Exo. (**D** and **E**) Immunohistochemical detection shows the expression of Ki67. ***P<0.001 *vs* NC. ^###^*P*<0.001 *vs.* CD133^+^-Exo. Star bar: 100 μm.

To determine the effects of CSC-derived exosomes on CRC tumorigenesis, we subcutaneously implanted SW480 cells into the flanks of nude mice and injected 5 mg of control or CSC-derived exosomes into the tail veins of the mice once every 5 days for 2 weeks. We found that exosomes from CD133^+^ cells accelerated tumor growth, as measured by increases in both volume and weight. However, downregulation of EIF3B reduced the growth-promoting effects of exosomes from CD133^+^ cells (Fig. **5A**-**C**). Immunohistochemical staining revealed that treatment with exosomes from CD133^+^ cells led to increased Ki-67 expression levels, while downregulation of EIF3B reduced this effect (Fig. **5D**-**E**).

## 4 DISCUSSION

CSCs are a self-renewing microsubpopulation within the tumor microenvironment that has been associated with increased resistance to radiation and other therapies. CSCs also control tumor heterogeneity, can alter the tumor microenvironment, and promote EMT [13-17]. Increasing evidence has confirmed that exosomes derived from cancer cells can establish a premetastatic niche in the liver, brain, and lungs [18, 19]. Specifically, CSC-derived exosomes associated with premetastatic niche formation and the biological identities of cancerous stromal elements may offer new targets with predictive value [20]. CSC-derived exosomes are associated with proliferation, migration, differentiation, angiogenesis, and metastasis through an enhanced process of self-renewal in which resistance to chemotherapy and radiotherapy may complicate cancer treatment [8]. Exosomes can facilitate crosstalk between CSCs and the tumor microenvironment [21]. The current investigation discovered that treatment with CD133^+^-derived exosomes enhanced the migration and proliferation of SW480 cells, as well as EMT.

High-throughput sequencing was used to identify genes and proteins delivered by exosomes, while qRT-PCR was used to examine differences in mRNA expression levels in exosomes derived from CD133^+^ cells and CD133^−^ cells. The CD133 protein is the predominant cell surface marker used to detect cancer cells exhibiting stem cell-like characteristics. CD133 alters common abnormal processes in colorectal cancer, such as the phosphoinositide 3-kinase/protein kinase B (AKT) and Wnt/β-catenin pathways. The study also found that CD133-containing microvesicles promote colorectal cancer progression by inducing tumor angiogenesis [22]. This study discovered that EIF3B expression was significantly higher in CD133^+^-derived exosomes compared to exosomes from CD133^−^ cells, indicating that Eukaryotic Translation Initiation Factor 3 Subunit B (EIF3B) may function to regulate the tumorigenic effects of CD133^+^ Exos on CRC cells.

## STUDY LIMITATIONS

EIF3 has 13 subunits and is the largest and most important EIF. EIF3B, one of its subunits, is generally considered the main scaffolding protein because it promotes the binding of other components with different affinities. Previous studies have demonstrated that aberrantly high expression levels of EIF3B in pancreatic cancer [23], chronic myelogenous leukemia [24], gastric cancer [25], non-small cell lung cancer [26], and osteosarcoma [27] are associated with unfavorable prognosis. EIF3B participates in cell proliferation, tumor invasion, and metastasis, and elevated expression of EIF3B is closely connected to the initiation and development of malignant tumors. Nevertheless, little is known regarding the role of EIF3B in CRC. The present investigation showed that knockdown of EIF3B decreased the proliferation- and migration-promoting effects of CD133^+^ Exos on SW480 cells. In addition, we demonstrated that treatment with CD133^+^ Exo promoted the tumorigenesis of SW480 cells *in vivo*.

## CONCLUSION

In summary, this study shows that treatment with exosomes from CD133^+^ cells promotes SW480 cell migration and proliferation. The mechanism study confirmed that it may be related to the EIF3B delivery. We hope that our study can provide a new theoretical basis for the clinical treatment of CRC. The limitation of this study was to clarified the regulatory mechanism of EIF3B to malignant progression of CRC.

## AUTHORS’ CONTRIBUTIONS

The authors confirm their contributions to this paper as follows: Y.W. was responsible for the analysis and interpretation of the results; X.L., X.H., and Z.W. contributed to drafting the manuscript; and J.Y. handled the visualization. All authors reviewed the results and approved the final version of the manuscript.

## Data Availability

The data and supportive information are available within the article.

## LIST OF ABBREVIATIONS

CRC: Colorectal Cancer
CSCs: Cancer Stem Cells
CD133^+^Exos: Exosomes Isolated from CD133^+^ Cells
qRT-PCR: Quantitative Real-time Polymerase Chain Reaction
CCK: Cell Counting Kit
EdU: Ethynyl-deoxyuridine
WB: Western Blot
EMT: Epithelial-To-Mesenchymal Transition
ALDH: Aldehyde Dehydrogenase
